# 5-Episinuleptolide Acetate, a Norcembranoidal Diterpene from the Formosan Soft Coral *Sinularia sp*., Induces Leukemia Cell Apoptosis through Hsp90 Inhibition

**DOI:** 10.3390/molecules18032924

**Published:** 2013-03-04

**Authors:** Kao-Jean Huang, Yu-Cheng Chen, Mohamed El-Shazly, Ying-Chi Du, Jui-Hsin Su, Chia-Wei Tsao, Wei-Hsuan Yen, Wen-Been Chang, Yin-Di Su, Yao-Tsung Yeh, Mei-Chin Lu

**Affiliations:** 1 Department of Life Science, National Dong Hwa University, Hualien, 974, Taiwan; 2 Graduate Institute of Marine Biotechnology, National Dong Hwa University, Pingtung 944, Taiwan; 3 Graduate Institute of Natural Products, College of Pharmacy, Kaohsiung Medical University, Kaohsiung 807, Taiwan; 4 Department of Pharmacognosy and Natural Products Chemistry, Faculty of Pharmacy, Ain-Shams University, Organization of African Unity Street, Abassia, Cairo 11566, Egypt; 5 National Museum of Marine Biology & Aquarium, Pingtung 944, Taiwan; 6 Institute of Marine Biodiversity and Evolution, National Dong Hwa University, Pingtung 944, Taiwan; 7 Department of Medical Laboratory Sciences and Biotechnology, Fooyin University, Kaohsiung 831, Taiwan

**Keywords:** 5-episinuleptolide acetate, apoptosis, soft coral, Hsp90, mitochondria, ROS, *Sinularia sp.*

## Abstract

5-Episinuleptolide acetate (5EPA), a cytotoxic norcembranoidal diterpene recently identified from the Formosan soft coral *Sinularia sp*., exhibited potent activity against the K562, Molt 4 and HL 60 cancer cell lines. The antiproliferative assay, as well as the annexin V-FITC/propidium iodide (PI) apoptotic assay, indicated that the HL 60 cell line is the most sensitive one towards 5EPA. This diterpenoid led to caspases -3, -8, and -9 activation as well as PARP cleavage. It also induced ROS generation, calcium accumulation and disruption of mitochondrial membrane potential. Additionally, the expression levels of Hsp90 protein and several client proteins were downregulated in response to 5EPA treatment. These results suggest that 5EPA’s cytotoxic effect on HL 60 cells may be attributed to the inhibition of Hsp90 as well as the induction of mitochondrial stress which finally results in apoptotic cell death.

## 1. Introduction

An increasing number of marine natural products isolated from soft corals of the genus *Sinularia* have been found to possess interesting biological activities, including immunoregulatory [1,2], anti-inflammatory [1], antifouling [3], neuroprotective [4], and cytotoxic effects [5,6,7,8,9]. Previous chemical studies of this genus revealed its richness in different classes of secondary metabolites, including sesquiterpenes, diterpenes, polyhydroxylated steroids, and polyamine compounds [10]. Among the isolated diterpenes are cembranoids, which play a vital role in the coral defense system against predators [11]. Cembranoids, macrocyclic diterpenoids, possess a wide diversity of chemical structures and biological activities. C_19_-Norcembranoids is a subclass of C_20_-cembranoids, which differs from the parent class in the loss of the methyl substitution at C4 [12]. C_19_-Norcembranoids have attracted attention due to their potent cytotoxic and antiinflammatory activities, however, most of the previous reports have focused on the identification of the cancer cell lines susceptible to C_19_-norcembranoids without studying their cytotoxic mechanism of action. Naturally, revealing the molecular target of any cytotoxic agent is crucial for efficient structure activity relationship studies, as well as potential drug lead development. 

**Figure 1:**
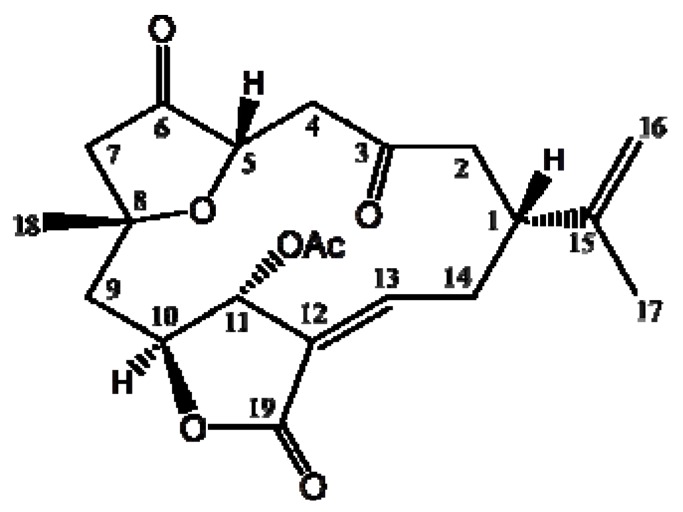
Chemical structure of 5-episinuleptolide acetate (5EPA), a norcembranoidal diterpene isolated from the Formosan soft coral *Sinularia sp*.

In our previous report we isolated a macrocyclic 3(2*H*)-furanone-based norcembranoid derivative, 5-episinuleptolide acetate (5EPA, Figure 1), from a Formosan soft coral identified as *Sinularia* *sp.* [8]. This derivative possesses a 14-membered carbocyclic ring backbone which encloses a furan and butenolide rings. It is suggested that the formation of this derivative and other similar structures involves the intramolecular cyclization of geranylgeranyl diphosphate leading to a 14-membered ring hydrocarbon intermediate, followed by selective enzymatic oxidations and ring closures, to yield the furan and butenolide ring-containing structure which further loses the substitution at C4 to produce the main C_19_-norcembranoid nucleus. In our previous study, we evaluated its cytotoxic activity against different cancer cell lines and found that this diterpenoid exhibited potent cytotoxic activity against leukemia cancer cell lines (K562 and Molt 4), in which it showed comparable IC_50_ values to the positive control, doxorubicin. The unique structure, as well as the potent cytotoxic activity of 5EPA against leukemia cancer cell lines, suggested its potential as an anti-leukemia drug lead and called for the identification of its molecular target.

Heat shock protein 90 is an essential and abundant chaperone which comprises about 1%–2% of the total proteins in the cell [13]. It facilitates folding of its client proteins that are involved in signal transduction, protein trafficking, receptor maturation and innate and adaptive immunity. Recent findings suggested a connection between Hsp90 and tumor cytotoxic drug resistance. It was found that cancer cells use the Hsp90 chaperone machinery to facilitate the function of numerous oncoproteins, thus promoting angiogenesis, survival, and resistance to apoptosis [14,15]. Consequently, Hsp90 has emerged as an interesting molecular target for developing new anticancer agents, especially against hematological malignancies and leukemia stem cells [16]. Recently, several Hsp90 inhibitors, such as tanespimycin, alvespimycin, and IPI-504, have been evaluated in clinical trials for the treatment of leukemia, melanoma, breast and lung cancers [17,18,19,20]. Based on the aforementioned remarks, we speculated that 5EPA may possess inhibitory effect on Hsp90, which we investigate in the current paper.

## 2. Results and Discussion

### 2.1. 5EPA is a Potential Inhibitor of Cell Growth and Inducer of Apoptosis in Leukemia Cells

**Figure 2:**
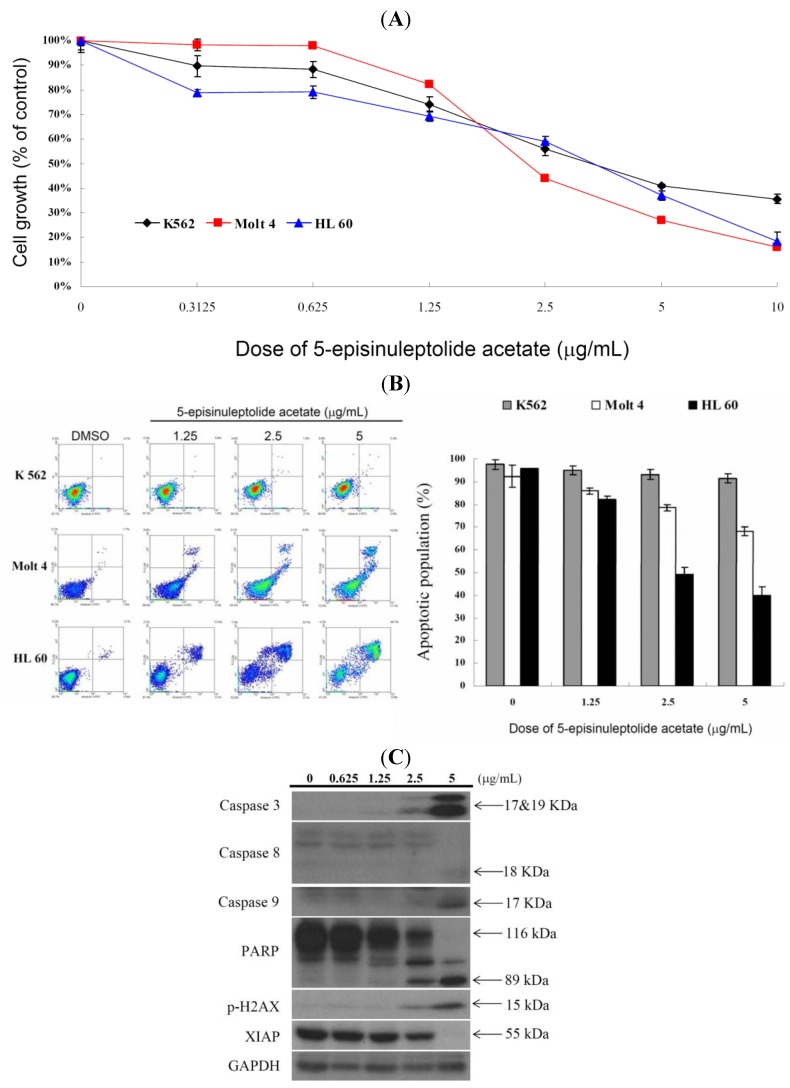
Cytotoxic and apoptotic effects of 5EPA on K 562, Molt 4 and HL 60 leukemia cells. (**A**) Leukemia cells were treated with varying concentrations of 5EPA for 24 h. Cell growth was assayed by MTT method; (**B**) Leukemia cell lines were treated with varying concentrations of 5EPA for 24 h, then labeled with annexin V-FITC and PI (propidium iodide) and analyzed with flow cytometry; (**C**) HL 60 cells were exposed to different doses of 5EPA for 24 h, collected and the expression of caspases activation, PARP cleavage and H2A.X phosphorylation were determined utilizing western blotting assay.

In our previous report, we found that 5EPA (Figure 1) exhibited potent cytotoxic activity against a number of cancer cell lines such as K562, Molt 4 cells, HCT-116, DLD-1 cells, t-47D and MDA-MB-231 cells, with IC_50_ values of 0.67, 0.59, 4.09, 0.92, 3.09, and 2.95 μg/mL, respectively, after 72 h. Leukemia cell lines (K562 and Molt 4) and a colon cancer cell line (DLD-1) were the cell lines most sensitive to the cytotoxic effects of 5EPA [8]. This encouraged us to expand our cytotoxicity study aiming to reveal the mechanism of action of 5EPA against leukemia cancer cell lines, which we pursue in the current study. Initially, we determined the IC_50_ values of 5EPA against K562, Molt 4 and HL 60 cells after 24 h and found the IC_50_ to be 4.09, 3.21 and 2.53 μg/mL, respectively (Figure 2A). Then we tried to evaluate if the cytotoxic activity of 5EPA is associated with apoptosis, by examining 5EPA’s effect on cells stained with annexin V-FITC and propidium iodide (PI). As shown in Figure 2B, treatment with 5EPA at concentrations of 0, 1.25, 2.5, and 5 μg/mL, increased the percentages of annexin-positive cells from 5.6% to 8.5%, 31.8% and 60.2% in K562, Molt 4 and HL 60 cells, respectively. The cytotoxic and apoptotic results indicated that HL 60 showed the highest sensitivity to 5EPA treatment and thus was selected as the optimum cell line to reveal 5EPA mechanism of action. To assess the mechanism of 5EPA-induced apoptosis, the levels of apoptosis-regulated proteins in HL 60 cells after treatment with different doses of 5EPA for 24 h, were evaluated. The results shown in Figure 2C from the western blotting assay, suggested that 5EPA treatment resulted in a substantial up-regulation of caspases -3, -8 and -9; cleavage of PARP (89 KDa); and H2A.X phosphorylation; as well as down-regulation of XIAP, a caspase inhibitor.

### 2.2. 5EPA Treatment Induced HL 60 Cells Mitochondrial Stress

**Figure 3:**
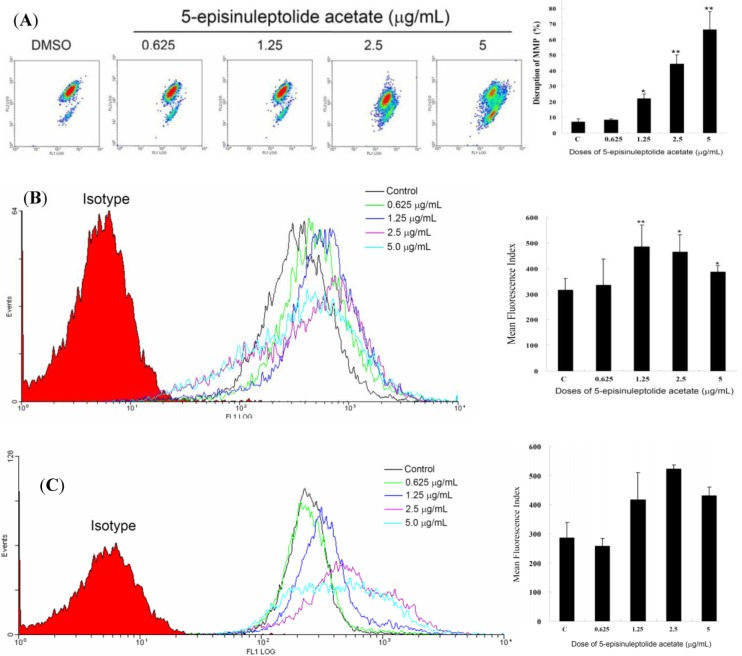
Flow cytometric results showing the effect of using different concentrations of 5EPA (0.625, 1.25, 2.5 and 5 μg/mL) on the disruption of mitochondrial membrane potential (MMP), generation of ROS, and accumulation of calcium in HL 60 cells. (**A**) Disruption of MMP; (**B**) ROS production; (**C**) Ca^2+^ release. The results indicated an increase in ROS production and Ca^2+^ release reaching the maximum levels at 1.25 and 2.5 μg/mL, respectively followed by a gradual decrease at higher doses. On the other hand, 5EPA treatment led a gradual increase in disruption of MMP when compared to the mean fluorescent index (MFI) of the untreated control. Results are presented as mean ± SD of three independent experiments (* *p* < 0.05; ** *p* < 0.001).

To examine if the antiproliferative and apoptotic effects of 5EPA involved mitochondrial stress in HL 60 cells, flow cytometric assay with various fluorescent dyes was utilized. Different concentrations of 5EPA (0, 0.625, 1.25, 2.5, and 5 µg/mL) for 24 h were used and the change of mitochondrial membrane potential (MMP), reactive oxygen species (ROS) production, and Ca^2+^ release were analyzed. Treatment with 5EPA (1.25, 2.5 or 5 µg/mL), led to 22.03, 44.32 and 66.17% disruption of MMP, as detected with JC-1 cationic dye in HL 60 cells (Figure 3A). The generation of ROS was examined with a carboxy derivative of fluorescein dye, carboxy-H_2_DCFDA. As shown in Figure 3B, treatment with 5EPA at 1.25, 2.5 and 5 µg/mL induced 0.56-, 0.47-, and 0.23-fold increase in the generation of ROS, respectively, as compared to the mean fluorescent index (MFI) of the control. For the determination of intracellular Ca^2+^ release, a fluorescent calcium indicator, Fluo 3, was used. The flow cytometric results showed that the treatment with 5EPA at different concentrations (1.25, 2.5 and 5 µg/mL) induced 0.46-, 0.82-, and 0.51-fold increase in the intracellular Ca^2+^ accumulation, respectively as compared to MFI of the control (Figure 3C).

### 2.3. 5EPA Induced HL 60 Cells Apoptosis Mediated Inhibition of Hsp90 and Several Client Proteins

**Figure 4:**
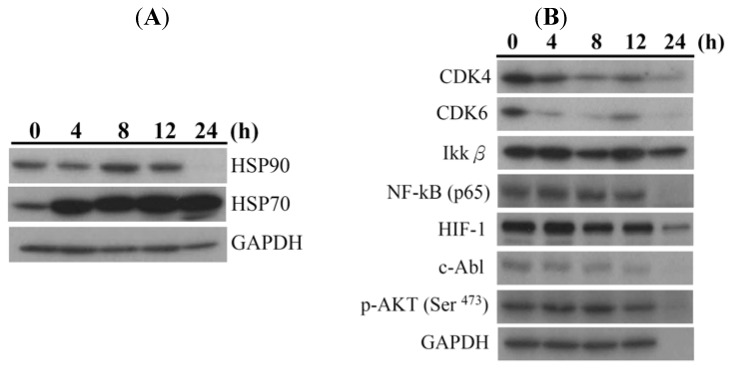
Apoptosis-induced by 5EPA is mediated through the inhibition of Hsp90 and its client proteins in HL 60 cells. Cells were treated with 2.5 μg/mL of 5EPA for 4, 8, 12 and 24 h, respectively. Cells were harvested and lysates were prepared and subjected to SDS-PAGE followed by immunoblotting. Western blotting analysis of the expression of (**A**) Hsp70 and 90 proteins; (**B**) several Hsp90 client proteins in HL 60 cells. GAPDH was used as an internal control to show the equal loading of the proteins.

The effect of 5EPA on Hsp90 and its client proteins was investigated aiming to reveal any correlation between 5EPA’s cytotoxic effects and the inhibition of Hsp90. Figure 4A demonstrates the effect of 5EPA treatment on the expression of Hsp90 and Hsp70, a co-chaperone which assists Hsp90 in its chaperone activity. It was found that the use of 5EPA (2.5 μg/mL) resulted in a significant increase in Hsp70 protein level but a decrease in Hsp90 protein level in a time-dependent manner. Moreover, 5EPA significantly decreased Hsp90 client proteins levels such as c-Abl (oncoprotein), Akt (apoptotic proteins), CDK4 and 6 (cell cycle regulatory proteins), as well as HIF 1 and NFκB (transcription factors) as shown in Figure 4B. These effects are in compliance with the effects of other Hsp90 inhibitors such as alvespimycin and BIIB021 which induce apoptosis, expression of Hsp70 protein levels and inhibit Hsp90 client proteins [16,21].

## 3. Experimental

### 3.1. Bioassays Materials

RPMI 1640 medium, foetal calf serum (FCS), trypan blue, penicillin G, and streptomycin were obtained from Gibco BRL (Gaithersburg, MD, USA). 3-(4,5-dimethylthiazol-2-yl)-2,5-diphenyl-tetrazolium bromide (MTT), dimethylsulfoxide (DMSO), and all other chemicals were purchased from Sigma-Aldrich (St. Louis, MO, USA). Antibodies against c-Abl, caspases -3, -8, and -9, and 6, p-Akt (Ser^473^) and PARP were purchased from Cell Signalling Technologies (Beverly, MA, USA). Antibodies of Ikkβ, GAPDH, XIAP, CDK4, and NFκB (p65) were obtained from Santa Cruz Biotechnology (Santa Cruz, CA, USA). HIF 1, Hsp70 and 90 were from Enzo Life Science International, INC. JC-1 cationic dye, the carboxy derivative of fluorescein (carboxy-H_2_DCFDA), and fluorescent calcium indicator (Fluo 3) were purchased from Molecular Probes and Invitrogen detection technologies (Carlsbad, CA, USA). Anti-mouse and rabbit IgG peroxidase-conjugated secondary antibody were purchased from Pierce (Rockford, IL, USA). Hybond ECL transfer membrane and ECL western blotting detection kits were obtained from Amersham Life Sciences (Amersham, UK).

### 3.2. Preparation of 5-Episinuleptolide Acetate (5EPA) Stock Solution

5EPA was isolated and purified from the coral, and its chemical structure was confirmed through comparison with the reported spectral data (^1^H-NMR, ^13^C-NMR, and 2D NMR) [8]. 5EPA was dissolved in DMSO at a concentration of 10 μg/mL and diluted before use.

### 3.3. MTT Antiproliferative Assay

Cells were seeded at 4 × 10^4^ per well in 96-well culture plates before treatment with different concentrations of the tested compound. After treatment for 24, 48, or 72 h, the cytotoxicity of the tested compound was determined using MTT cell proliferation assay (thiazolyl blue tetrazolium bromide, Sigma-M2128). Light absorbance values (OD = OD_570_ − OD_620_) were recorded at wavelengths of 570 and 620 nm using an ELISA reader for calculating the 50% inhibitory concentration (IC_50_), *i.e.*, the cell concentration at which the light absorbance value of the experimental group is half that of the control group. These results were expressed as a percentage of the control ± SD established from n = 4 wells per one experiment from three separate experiments.

### 3.4. Annexin V/PI Apoptosis Assay

The externalization of phosphatidylserine (PS) and membrane integrity were quantified using an annexin V- FITC staining kit. In brief, 10^6^ cells were grown in 35 mm diameter plates and were labeled with annexin V-FITC (10 µg/mL) and PI (20 µg/mL) prior to harvesting. After labeling, all plates were washed with a binding buffer and harvested. Cells were resuspended in the binding buffer at a concentration of 2 × 10^5^ cells/mL before analysis by flow cytometer FACS-Calibur (Becton-Dickinson, San Jose, CA, USA) and CellQuest software. Approximately 10,000 cells were counted for each determination.

### 3.5. Determination of ROS Generation, Ca^2+^ Release, and MMP Disruption

MMP disruption, ROS generation and intracellular Ca^2+^ release were detected with JC-1 cationic dye (5 μg/mL), the carboxy derivative of fluorescein (carboxy-H_2_DCFDA, 1 mM), and the fluorescent calcium indicator (Fluo 3, 5 mM), respectively. In brief, treated cells were labeled with a specific fluorescent dye for 30 min. After labeling, cells were washed with PBS and resuspended in PBS at a concentration of 1 × 10^6^ cells/mL before analysis by flow cytometry.

### 3.6. Western Blotting Analysis

Cell lysates were prepared by treating the cells for 30 min in RIPA lysis buffer, 1% Nonidet P-40, 0.5% sodium deoxycholate, 0.1% sodium dodecyl sulphate (SDS), 1 mM sodium orthovanadate, 100 µg/mL phenylmethylsulfonyl fluoride and 30 µg/mL aprotinin) (all chemicals were obtained from Sigma). The lysates were centrifuged at 20,000 × *g* for 30 min, and the protein concentration in the supernatant was determined using a BCA protein assay kit (Pierce, Rockford, IL, USA). Equal amounts of proteins were respectively separated by 7.5%, 10% or 12% of SDS-polyacrylamide gel electrophoresis and then were electrotransferred to a PVDF membrane. The membrane was blocked with a solution containing 5% non-fat dry milk TBST buffer (20 mM Tris-HCl, pH 7.4, 150 mM NaCl and 0.1% Tween 20) for 1 h and washed with TBST buffer. The protein expressions were monitored by immunoblotting using specific antibodies. These proteins were detected by an enhanced chemiluminescence kit (Pierce).

### 3.7. Statistics

The results were expressed as mean ± standard deviation (SD). Comparison in each experiment was performed using an unpaired Student’s *t*-test and a *p* value of less than 0.05 was considered to be statistically significant.

## 4. Conclusions

Our results suggest that the use of 5-episinuleptolide acetate induced ROS generation, calcium accumulation and MMP disruption, as well as apoptosis in HL 60 cells. Additionally, 5EPA (at 2.5 μg/mL) inhibited Hsp90 protein expression levels, but induced Hsp70 protein expression levels as demonstrated by western blotting analysis. Moreover, 5EPA treatment down-regulated the expression of the Hsp90 client proteins including anti-apoptotic protein (XIAP and Akt), cell cycle regulatory proteins (CDK4 and 6), IκB kinase (IKKβ and NFκB), transcription factor (HIF 1), and oncogenic protein (c-Abl). Hsp90 is a highly abundant chaperone and possesses antiapoptotic effect through directly binding to Apaf-1 and recruiting procaspase 9 as well as interacting with phosphorylated Akt to phosphorylate IκB kinase, which leads to cell survival [22]. The current work clearly supports the potential application of 5EPA as Hsp90 inhibitor for leukemia therapy. 
